# Biological therapy for severe asthma

**DOI:** 10.1186/s40733-021-00078-w

**Published:** 2021-08-13

**Authors:** Silvano Dragonieri, Giovanna Elisiana Carpagnano

**Affiliations:** Department of Respiratory Diseases, University of Bari “Aldo Moro”, Piazza Giulio Cesare 11, 70124 Bari, Italy

**Keywords:** Severe asthma, Biologics, Biological therapy

## Abstract

Around 5–10% of the total asthmatic population suffer from severe or uncontrolled asthma, which is associated with increased mortality and hospitalization, increased health care burden and worse quality of life. In the last few years, new drugs have been launched and several asthma phenotypes according to definite biomarkers have been identified. In particular, therapy with biologics has revolutionized the management and the treatment of severe asthma, showing high therapeutic efficacy associated with significant clinical benefits. To date, four types of biologics are licensed for severe asthma, i.e. omalizumab (anti-immunoglobulin E) antibody, mepolizumab and reslizumab (anti-interleukin [IL]-5antibody), benralizumab (anti-IL-5 receptor a antibody) and dupilumab (anti-IL-4 receptor alpha antibody). The aim of this article was to review the biologic therapies currently available for the treatment of severe asthma, in order to help physicians to choose the most suitable biologic agent for their asthmatic patients.

## Background

Since the beginning of this millennium, asthma assessment and management have been revolutionized. While some new therapeutic approaches have been suggested for mild asthmatics, the most relevant changes have occurred in severe asthma. Severe asthma accounts for the 5–10% of the global asthma population, with 3 to 5% being uncontrolled despite adherence to therapy and proper use of inhalers [1]. These subjects cannot achieve symptoms control despite maximal therapy with inhaled corticosteroids (ICS) and, quite often, maintenance oral corticosteroids (OCS) are necessary in an endeavor to avoid life-threatening exacerbations [2]. Although OCS courses remain essential for the management of acute exacerbations, their recurrent or continuous usage is associated with several complications, such as an increased risk of developing osteoporotic fractures and pneumonia [3]. Moreover, other conditions including cardiovascular and cerebrovascular events, renal dysfunction, diabetes mellitus type 2, humor alterations, obesity and sleep apneas are known to be associated with systemic corticosteroid exposure [3]. Additionally, many patients remain poorly controlled and show recurrent exacerbations despite a strict adherence to therapy [4].

The recent advances in our knowledge of the etiopathological mechanisms of different phenotypes and endotypes of severe asthma gave us very innovative therapies, such as biological drugs for severe asthma. These medications are mostly directed against molecules involved in the type 2 inflammatory pathway, thus modifying the natural course of the disease by reducing airways inflammation without the collateral damage associated with corticosteroids. Based on the above, the aim of this article was to review the biologic therapies currently available for the treatment of severe asthma, in order to help physicians to choose the most suitable biologic agent for their asthmatic patients.

### Licensed medications for severe asthma

To date, there are five biologic molecules officially approved for use in selected severe asthmatic patients. The first of these is omalizumab, an anti-IgE monoclonal antibody acting through various mechanisms on allergic pathways (Table 1). Three more biologics for asthma, belonging to a different class, have been approved, i.e. mepolizumab, reslizumab and benralizumab. They all target the interleukin-5 (IL-5) pathway with the first two targeting the interleukin itself and the last one its receptor. Finally, dupilumab is a monoclonal antibody against the receptor of interleukin-4 (IL-4) which blocks the signaling pathways of IL-4 and IL-13.

**Table 1:** Licensed biologics for severe asthma

DRUG NAME	TARGET	MODE OF ADMINISTRATION	CRITERIA OF PRESCRIPTION	AGE INDICATION	DOSING INTERVAL	DOSAGE
Omalizumab	IgE	Subcutaneous injection	high blood IgE; sensititazion to perennial allergen	≥6 years	14 days or 28 days	75 mg to 600 mg (based on kg and tot IgE)
Mepolizumab	IL-5	Subcutaneous injection	high blood eosinophils	≥6 years	28 days	100 mg
Reslizumab	IL-5	Intravenous injection	high blood eosinophils	≥ 18 years	28 days	3 mg/kg
Benralizumab	IL-5 receptor a	Subcutaneous injection	high blood eosinophils	≥ 18 years	28 days (56 days after 3 months)	30 mg
Dupilumab	IL-4 receptor alpha	Subcutaneous injection	high blood eosinophils and/or raised FeNO	≥12 years	14 days	200 mg to 300 mg (based on comorbidities)

## BIOLOGICS TARGETING IgE

### OMALIZUMAB

Omalizumab was the first targeted biologic therapy developed and licensed for severe asthma, being approved by the Food and Drugs Administration in 2003 [5]. It is a recombinant monoclonal Antibody which binds to IgE, thereby lowering blood IgE levels of up to 99% [6]. Moreover, It decreases expression of IgE receptor FCRI on inflammatory cells such as mast cells and basophils, thus helping to both mitigate the allergic response and strengthen the antiviral immune response, finally leading to prevent asthma exacerbations [7]. Omalizumab is approved in adults and children above 6 years old with IgE-driven moderate-to-severe persistent allergic asthma which remains uncontrolled despite GINA step 4/5 treatment, high levels of blood IgE, and documented sensitization to a perennial allergen [8]. Its dosage varies according to patient’s bodyweight and circulating IgE levels and it is administered subcutaneously every 14 or 28 days [9]. Although not necessary from a safety point of view, it is advisable to re-evaluate patients after the initial 16 weeks of treatment to assess the drug efficacy before continuing with omalizumab therapy [8].

The efficacy and safety of omalizumab are nowadays unquestionably recognized, with numerous studies demonstrating that this biological is generally well-tolerated, with no serious adverse effects reported [10–15]. Common side effects include injection site or diffuse rash, fever, nose bleeding, joint pain, gastro-intestinal disturbances, headache, dizziness and cold symptoms [10–15]. A Cochrane systematic review assessing 25 randomized controlled trials in patients with allergic asthma showed the efficacy of omalizumab in reducing asthma exacerbations, hospitalizations, and inhaled corticosteroid dosage [10, 15–19].

During the last few years, a number of biomarkers for monitoring the efficacy of omalizumab therapy have been proposed, including total and antigen-specific IgE, blood eosinophil count and exhaled nitric oxide (FeNO) [20, 21]. Surprisingly, total IgE did not appear to be a reliable predictor of response to omalizumab therapy, evidencing that our knowledge on this field is still limited [21]. Peripheral blood eosinophil count ≥300 cells/mL are linked to higher asthma severity and to a better response to omalizumab [22, 23]. Furthermore, patients under omalizumab with higher blood eosinophil count have a higher chance to suffer from asthma exacerbations in case of omalizumab discontinuation [24]. Regarding FeNO, elevated values at baseline correlated with a better response to omalizumab with regard to exacerbations decrease [20, 25]. Likewise, elevated levels of FeNO after suspension of long-term therapy with omalizumab may be a predictor of successive exacerbations [24].

## Biologics targeting IL-5

IL-5 is a well-known regulator of the activation, differentiation, effector function, migration and survival and effector function of eosinophils [26]. Eosinophil levels associated with symptoms of asthma correlate with disease severity and increase the risk of asthma exacerbations, evidencing that this granulocyte type plays a key role in the pathophysiololgy of asthma [26]. Currently, licensed biologics against IL-5 pathways are mepolizumab, reslizumab, and benralizumab.

### MEPOLIZUMAB

Mepolizumab is a monoclonal antibody directed against IL-5 which has been approved as an add-on treatment for patients ≥6 years old in Europe and for patients ≥12 years old in the USA. Mepolizumab was the first anti-IL-5 antibody approved for the treatment of severe asthma by the Food and Drugs Administration in 2015. Eligible subjects are those with severe eosinophilic asthma that remains uncontrolled despite GINA step 4/5 therapy, with blood eosinophil count of ≥150 cells/μl during the first administration or ≥ 300 cells/μl in the previous year and with at least 2 asthma exacerbations requiring systemic steroid course in the past year [27, 28]. Mepolizumab is administered by a subcutaneous injection at a fixed dose of 100 mg every 28 days.

Several studies evaluating mepolizumab for uncontrolled eosinophilic asthma showed a markedly reduction with regard to number of exacerbations, systemic corticosteroid usage, emergency room accesses and hospital admissions, and a concurrent improvement of asthma controls and lung function parameters [29–33].

Furthermore, a number of studies revealed that mepolizumab has a positive long-term safety profile [34–36]. No reports of mepolizumab-associated anaphylaxis reactions were documented, as well as parasitic infections [34–36]. Common side effects include headache, injection site reaction, fatigue, flu symptoms, urinary tract infection, abdominal pain, itching, eczema, and muscle spasms [34–36].

Additionally, numerous investigations highlighted that the most important markers of response prediction to mepolizumab are the rate of previous exacerbation and baseline peripheral blood eosinophil count [29, 32, 37–39]. Indeed, a better clinical efficacy is directly proportional to a higher eosinophil count and to a higher rate of exacerbations [29, 32, 37–39]. Interestingly, mepolizumab effectiveness was not related to baseline IgE and to atopy [40, 41] and earlier treatment with omalizumab is not a predictor for mepolizumab efficacy [42–44].

There is a lack of consensus about the duration of treatment before evaluating the effectiveness of mepolizumab. Actually, the GINA statement suggests that a 4-month trial may be adequate [8], whereas the NICE guidelines recommend that mepolizumab should not be discontinued before 12 months of therapy and that drug-responsiveness should be assessed every year [45].

### RESLIZUMAB

Reslizumab is monoclonal antibody approved in 2016, which binds with high-affinity to IL-5 [46]. By an analogous mechanism of action to mepolizumab, reslizumab lowers circulating blood eosinophil levels [47]. It has been approved for patients ≥18 years old with severe eosinophilic asthma which remains uncontrolled despite therapy with high-doses of ICS plus another inhaler. Reslizumab is indicated in patients with ≥400 eosinophils/μl and history of asthma exacerbations in the previous 12 months [48, 49]. Reslizumab is administered intravenously every 28 days at a weight-based dose of 3 mg/kg.

Similarly to mepolizumab, studies assessing reslizumab have shown a decreased number of asthma exacerbations and improved asthma control and lung function parameters in subjects with high blood eosinophil levels [47, 50].

The safety profile of reslizumab has been evaluated for up to 24 months, revealing minor adverse effects without any reports of parasitic and opportunistic infections [51]. Most frequent side effects include cough, dizziness, itching, skin rash and fatigue [51].

However, despite its proven excellent clinical efficacy, intravenous formulation has a significant impact on the ease of administration compared to mepolizumab and/or benralizumab. Studies using reslizumab showed unsatisfactory results, without significant improvements in terms of acute exacerbations reduction or OCS lowering [52].

### BENRALIZUMAB

Benralizumab is a monoclonal antibody approved in 2017 and directed against IL-5 receptor a (IL-5Ra) which induces eosinophil apoptosis via the antibody-dependent cell-mediated cytotoxicity (ADCC) involving natural killer cells, leading to peripheral blood eosinophil depletion [53, 54]. Benralizumab acts like a competitive inhibitor to IL-5, binding with higher affinity to the a-subunit of IL-5Ra, which is expressed on mature (and precursors) eosinophils and basophils [55].

This biologic drug is licensed as an add-on treatment for uncontrolled severe eosinophilic asthma in patients ≥18 years with ≥300 blood eosinophils/μl [56, 57]. A 30 mg dose of benralizumab is injected subcutaneously every 28 days for the first 3 administrations and afterwards every 56 days.

Large studies evaluating benralizumab in patients with moderate to severe asthma have shown a decrease in exacerbations number, improved lung function, and reduced use of OCS [53, 54, 58]. Combined analysis of these investigation have revealed that the best predictors of response to benralizumab are adult-onset asthma, more than 3 exacerbations in the previous year, nasal polyposis and pre-bronchodilator FVC < 65% of predicted [53, 54, 58].. The most common adverse effect were fever after the first injection, headache and pharyngitis [53, 54, 58].

Interestingly, based on its mechanism, benralizumab almost completely depletes blood eosinophils within 24 h of administration and a total depletion of airway eosinophils compared to that caused by mepolizumab [59, 60]. Likewise, nasal eosinophils were totally suppressed after 6 months of therapy with benralizumab [61].

Recently, some concerns have been raised about the theoretical risks following an eosinophil depletion, especially with respect to host defense. However, these warnings were not confirmed, since it appears that there is adequate redundancy within human immune apparatus, which is not impaired by eosinophils depletion [62].

## Biologics targeting IL-4 and IL-13

IL-4 and IL-13 are two interleukins which regulate and drive Type-2 inflammation. IL-4 increases the Th-2 cell population and B-cell isotype rearrangement of IgE as well as promoting eosinophilic transmigration through endothelium, whereas IL-13 plays an important role in asthma by promoting airway hyperresponsiveness, mucus secretion and airway remodeling [63, 64]. Thus far, the only licensed drug acting on the two aforementioned ILs is dupilumab.

### DUPILUMAB

Dupilumab is a monoclonal antibody approved in 2018 which binds to the IL-4 receptor alpha-subunit, mutual to IL-4 and IL-13 receptors and inhibits both IL-4 and IL-13 pathways. Dupilumab is licensed as an add-on maintenance therapy in asthmatic patients GINA step 4/5 ≥ 12 years with type 2 inflammation characterized by increased blood eosinophils and/or raised FeNO. Dupilumab is administered subcutaneously at a starting dose of two injections of 200 mg each (total 400 mg), followed by one injection of 200 mg every 14 days, or at a starting dose of 600 mg (two injections of 300 mg each) followed by 300 mg every 14 days. The latter regimen is recommended for asthmatic subjects strictly dependent from OCS or with atopic dermatitis [65]. Dupilumab is also indicated for moderate to severe atopic dermatitis and for nasal polyposis.

A number of studies have demonstrated that therapy with dupilumab in severe asthmatics lowers the number of asthma exacerbations, improves lung function parameters and asthma control test scores, and lowers the use of OCS, irrespective of peripheral blood eosinophil count [66–69]. Indeed, a transitory increase of blood eosinophilia at the beginning of treatment with dupilumab has been observed although it may be due to blocked migration into tissues rather than hyperproduction [69]. Furthermore, reduced levels of T2 inflammation markers, including FeNO, serum levels of eotaxin-3, periostin and thymus and activation regulated chemokine (TARC) and total IgE, may serve as parameters for monitoring the efficacy of therapy with dupilumab [66–69]. The most common adverse reactions were injection site reactions, various types of infections, conjunctivitis and related conditions [66–69].

## Biologics under development

Research for next-generation biologics is ongoing. Currently, other effector molecules are under the spotlight as new targets for perspective biological therapies, particularly the so-called alarmins [70]. These molecules are released by the airway epithelium against the harmful actions of germs, pollutants, allergens and cigarette smoke.

Tezepelumab is a human monoclonal antibody which binds to thymic stromal lymphopoietin (TSLP), an epithelium-derived alarmin that plays a relevant role in the pathogenesis of asthma, being an upstream effector T2-high pathobiologic pathways [71–73]. With the presence of tezepelumab, TLSP cannot bind to its receptor [74] hence inhibiting downstream signaling. A number of phase 2 and 3 trials have clearly shown that patients with severe uncontrolled asthma who received tezepelumab had fewer exacerbations and better lung function, asthma control, and health-related quality of life than those who received placebo [75, 76]. Concerning its safety profile, neither investigational tezepelumab-related anaphylactic reactions nor the detection of neutralizing antibodies were reported [75, 76]. To date, license application for tezepelumab has been accepted and granted Priority Review for the treatment of asthma from the US Food and Drug Administration, whose regulatory decision is expected during the first quarter of 2022.

Ipetekimab is a monoclonal antibody targeting IL-33, another alarmin which associates with TSLP leading to an activation of T2-high inflammatory pathway in asthma [77]. Phase 2 studies with this biologic are ongoing, however preliminary results did not show adequate efficacy in severe asthmatics when associated with dupilumab or vs dupilumab alone [70].

Moreover, Tralokinumab and lebrokizumab are monoclonal antibodies both targeting IL-13 alone with disappointing results of phase 3 studies in terms of exacerbations reduction and OCS sparing in severe asthmatics [78].

Finally, regarding Th2-low asthma, mainly characterized by a neutrophilic airways inflammation, efforts are focusing on its pathogenic cascade involving cytokines such as IL-1beta, IL-17 and IL-23. Several monoclonal antibodies against the aforementioned interleukins such as canakinumab (anti IL-1beta), brodalumab (anti IL-17 receptor) and risankizumab (anti IL-23) are under evaluation with phase 1–2 trials showing controversial results [79–81].

### Which biologic should I choose for my asthmatic patient?

**Fig. 1:**
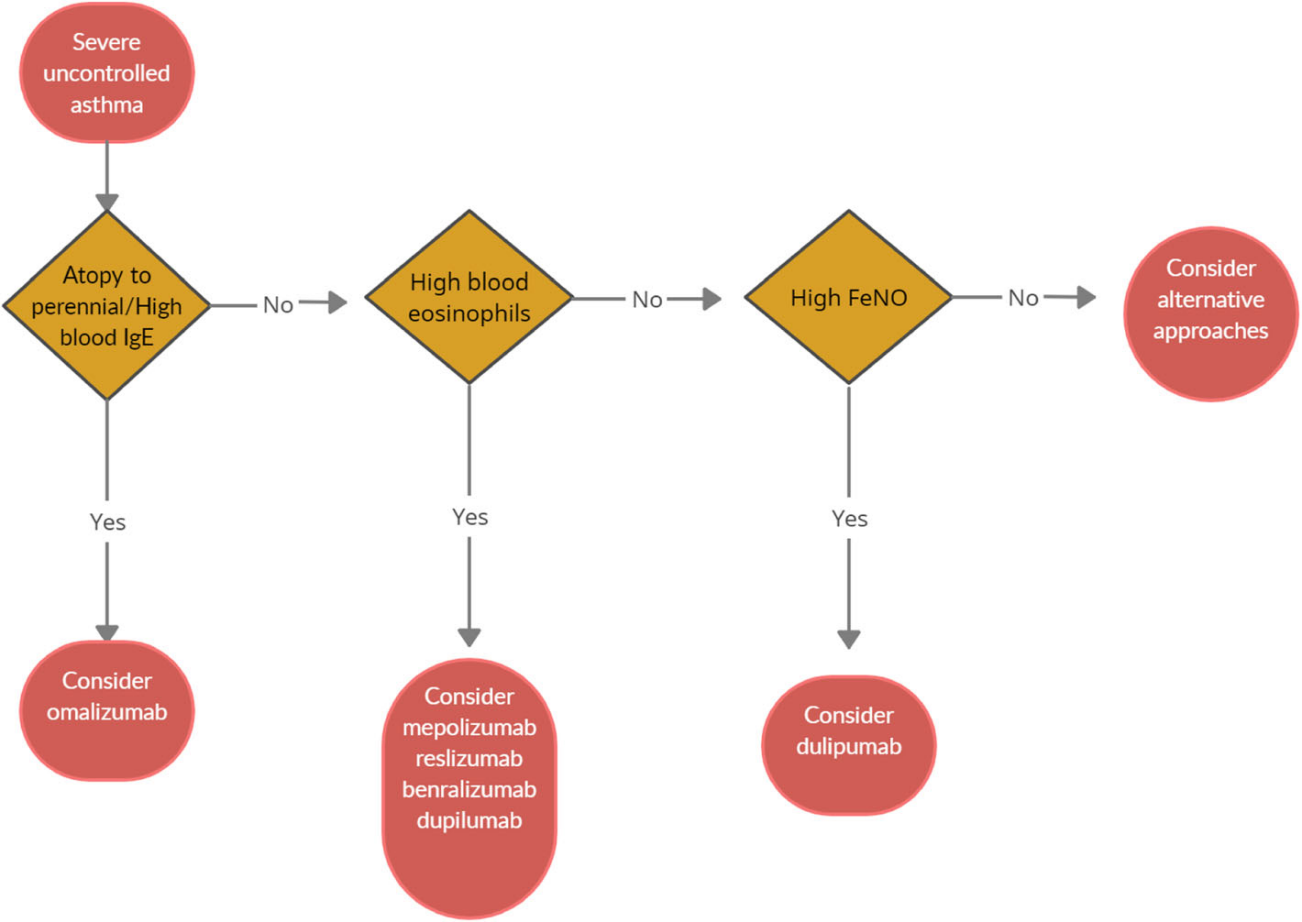
Algorithm for Selecting Ideal Biologic Treatment for severe uncontrolled asthma

When choosing a biologic medication for their patients with severe uncontrolled asthma, clinicians should always take into account the asthma endotype, clinical biomarkers, and patient-focused aspects (Fig 1).

Omalizumab should always be the first biological option in allergic non-eosinophilic severe asthmatics, with high levels of blood IgE, and with at least a documented positivity to a perennial aeroallergen. Contrariwise, patients with a non-allergic eosinophilic phenotype should be treated with an anti-IL-5 biological drug. Finally, anti- IL-4/IL-13 should be reserved to patients with severe eosinophilic type 2 asthma OCS dependent [8].

Given to the a lack of comparison studies, to date there are no recommendations about the selection of appropriate anti IL-5 biologic drug among those available. Hence, the choice is empirical and possibly shared between physician and patient.

According to GINA guidelines, a (at least) 4-month trial should be carried to evaluate asthma control. In the event of poor asthma control, a switch to a different biological treatment can be attempted if the patient meets the eligibility criteria.

Nevertheless, the right time and the right modality of switching from one biologic to another and the treatment time are still unknown. Large studies focused on biological drug switch in patients with severe asthma are ongoing and will help physicians to ease therapeutic strategies.

## Conclusions

Severe asthma accounts for a small proportion of total asthma cases, but impose a heavy burden on health care system. Recent revelations of the T2 inflammatory pathways and the development of monoclonal antibodies acting on the T2 cascade has completely revolutionized the management of severe asthma, by introducing new, life-improving treatment options for this class of patients. This paves the way for a biomarker-driven personalized medicine. Strictly following GINA recommendations, the categorization of T2 molecular targets has allowed the identification of patients with severe asthma who would likely respond to specific biological molecules. However, the most suitable biological option for severe asthmatics with overlapping phenotypes is still unclear, thus requiring further discriminatory and predicting biomarkers which may allow a better patient selection.

## Data Availability

Not applicable.

## Abbreviations

IL: interleukin
ICS: inhaled corticosteroids
OCS: oral corticosteroids
IgE: immunoglobulin E
FeNO: fractional exhaled nitric oxide
FVC: forced vital capacity
T2: type 2
